# The complete chloroplast genome sequence of *Quercus phillyraeoides* (Fagaceae)

**DOI:** 10.1080/23802359.2020.1718029

**Published:** 2020-01-27

**Authors:** Chunping Xie, Dawei Liu, Chenghui Nan, Yanming Fang, Fulong Huang

**Affiliations:** a Institute of Criminal Science and Technology, Nanjing Forest Police College, Nanjing, China;; b College of Biology and the Environment, Nanjing Forestry University, Nanjing, China;; c Biodata Biotechnologies Co. Ltd, Hefei, China

**Keywords:** Chloroplast genome, *Quercus phillyraeoides*, phylogenetic analysis

## Abstract

*Quercus phillyraeoides* A. Gray is an evergreen oak species native to East Asia, and it plays a vital role in mountain ecosystems. In this study, we assembled the complete chloroplast genome of *Q. phillyraeoides* based on sequencing data. The genome was circular and 161,384 bp in length, consisting of a large single-copy region (90,617 bp), small single-copy region (19,035 bp), and two short inverted repeat regions (25,866 bp). We found that the cp genome encodes for 131 genes, including 85 protein-coding genes (PCGs), 8 rRNA genes, and 37 tRNA genes. The results of the phylogenetic analysis of the complete cp genome sequence illustrated that *Q. phillyraeoides* is a member of the section *Quercus* and it is most closely related to *Q. tarokoensis*.

The evergreen oak species *Quercus phillyraeoides* A. Gray is native to China and Japan. In China, it is widely distributed in the subtropical area, and it grows at an altitude range from 200 to 1700 m a.s.l. (Xie et al. 2011). As one of the dominant trees of subtropical mixed mesophytic forests, it is of high ecological significance for preserving mountain ecosystems, including erosion control in montane forests and providing habitats and food for different species. It is also an economically important tree species for producing excellent charcoal without smoke, which is particularly popular in Japan and Korea. However, few studies were focused on genomic resources of this species (Liu et al. 2013), which is why the plastid genome sequence of the species is still unknown. The aim of this research was to report the complete cp genome sequence of *Q. phillyraeoides* in order to provide the basis for future genetic studies on this and other relevant species.

Fresh leaves of *Q. phillyraeoides* were collected from a mature tree from the Nanjing Botanical Garden Memorial Sun Yat-Sen (NBG; Nanjing, China). The voucher specimen (accession no. 2019110101) was preserved at the Herbarium of Nanjing Forest Police College. Total genomic DNA was extracted and purified using the method described in Doyle and Doyle (1987). The whole genome sequencing on the BGISEQ-500 platform was performed by Biodata Biotechnology Co. Ltd (Hefei, China). In total, 35.09 M clean reads were obtained and assembled *de novo* using NOVOplasty 2.7.2 (Dierckxsens et al. 2016). Annotation was performed using DOGMA (Wyman et al., 2004). All tRNA sequences were confirmed using the web-based online tool tRNAScan-SE (Schattner et al. 2005) with default settings. The complete chloroplast genome sequence of *Q. phillyraeoides* was deposited in the GenBank with the accession number MN882701.

The plastome of *Q. phillyraeoides* was determined to comprise double-stranded, circular DNA of 161,384 bp containing two inverted repeat (IR) regions of 25,866 bp, each separated by large single-copy (LSC) and small single-copy (SSC) regions of 90,617 and 19,035 bp, respectively (NCBI acc. no. MN882701). The genome contained 131 genes, including 85 protein-coding genes, 37 tRNA genes, and eight rRNA genes. The six protein-coding genes, five tRNA genes, and four rRNA genes were duplicated in the IR region. Nineteen genes contained two exons and four genes (clpP, ycf3, and two rps12) contained three exons. The overall GC content of *Q. phillyraeoides* cp genome was 36.8%, and in LSC, SSC, and IR regions, this content is 34.6, 30.9, and 42.7%, respectively.

In order to investigate the taxonomic status of *Q. phillyraeoides*, whole chloroplast genomes of 32 Fagales species and two outgroup species (*Theobroma cacao* and *Populus trichocarpa*) were aligned using MAFFT version 7 (Katoh and Standley 2013). The maximum likelihood (ML) tree was reconstructed by FastTree version 2.1.10 (Price et al. 2010). The ML phylogenetic tree showed that among the species from the section *Quercus*, *Q. phillyraeoides* is most closely related to *Q. tarokoensis*, with bootstrap support values of 100% (Figure 1).

**Figure 1.**
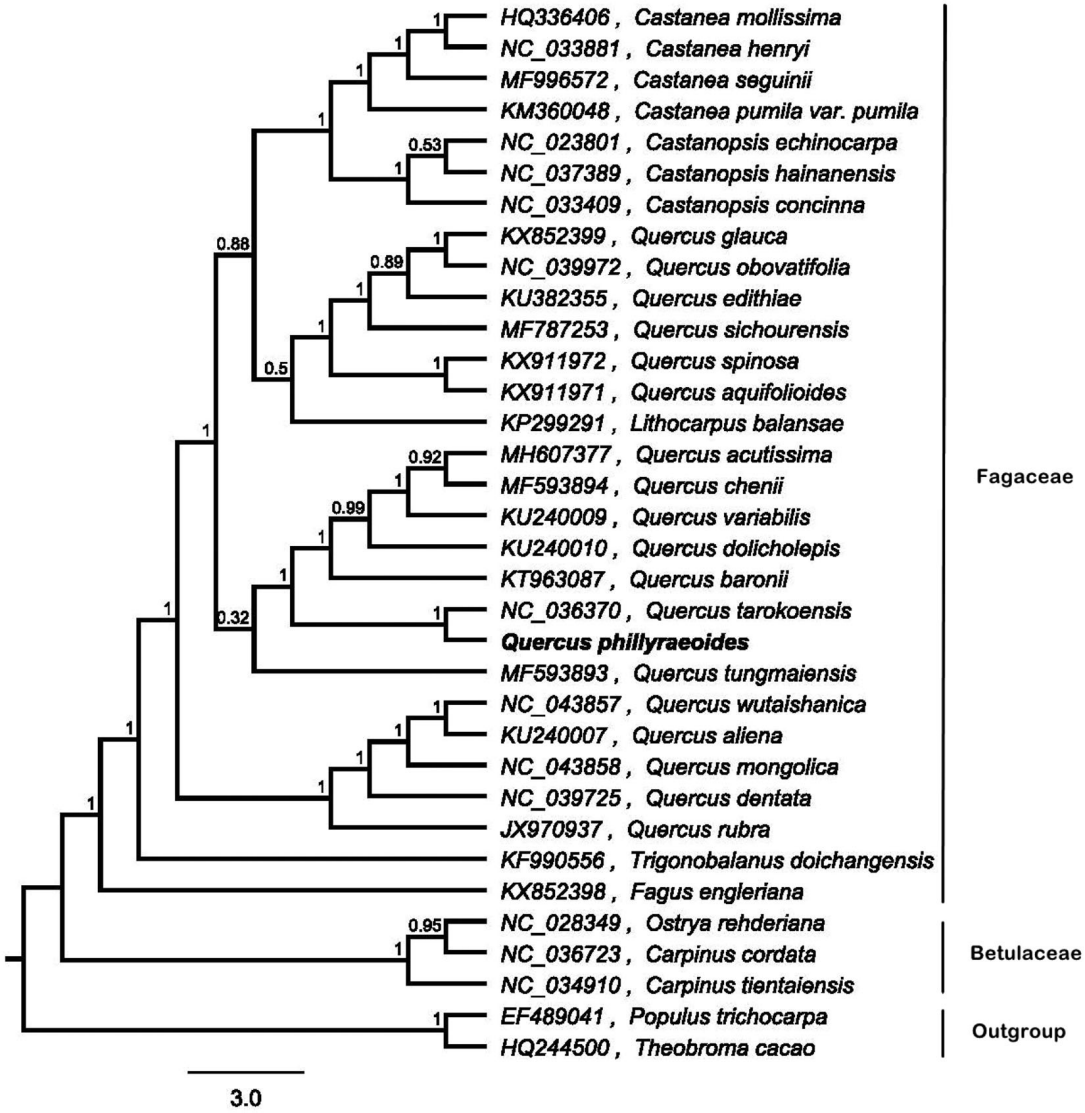
Maximum likelihood tree of 32 species of Fagales (Fagaceae and Betulaceae) and two outgroup taxa (*Theobroma cacao* and *Populus trichocarpa*) constructed in FastTree based on plastid genome sequences. Bootstrap support values are shown near the branching points.
